# Assessing the acceptability, reliability, and validity of the EORTC Quality of Life Questionnaire (QLQ-C30) in Kenyan cancer patients: a cross-sectional study

**DOI:** 10.1186/s41687-020-00275-w

**Published:** 2021-01-07

**Authors:** Jasmine Davda, Hillary Kibet, Emmah Achieng, Lawrence Atundo, Truphena Komen

**Affiliations:** 1 Certara Inc., Princeton, NJ USA; 2 International Cancer Institute, Eldoret, Kenya; 3 AMPATH, Eldoret, Kenya; 4 Moi Teaching and Referral Hospital, Eldoret, Kenya

**Keywords:** Cancer, Quality of life, Kenya, Validation, EORTC QLQ-C30

## Abstract

**Background:**

In oncology practice, eliciting the patient’s perspective on their quality of life (QOL) adds important information and value to their treatment and care. The European Organization for Research and Treatment of Cancer QOL questionnaire (EORTC QLQ-C30) is the most commonly used tool for this purpose but has not been validated in Kenya. The present study aimed to conduct a preliminary assessment of the QOL among Kenyan cancer patients and examine the psychometric properties of the tool in this population.

One hundred patients with heterogeneous types of cancer were enrolled in this cross-sectional study between July and August 2019. The EORTC QLQ-C30 questionnaire was administered to patients using either the English or Kiswahili official version. Descriptive statistics were used to assess patient demographics and clinical characteristics. The psychometric properties of the EORTC QLQ-C30 were evaluated in terms of acceptability, internal consistency, and construct validity using statistical software packages, STATA and SPSS.

**Results:**

The EORTC QLQ-C30 was found to be acceptable for use in our patient population as indicated by high compliance and low missing responses. Of the 100 patients, 66 were able to self-administer the questionnaire. The average time for completion was 13 min. Preliminary QOL assessment indicated an average QOL in Kenyan cancer patients (53 ± 27). Among the function scales, participants scored the lowest on the social function scale (51 ± 36) whereas among the symptom scales, participants scored the highest on the financial difficulties scale (79 ± 31). Cronbach’s alpha coefficient values ranged from 0.72–0.95, illustrating the reliability of the scales measured. Interscale correlations were statistically significant (*p* < 0.05), indicating clinical validity of the data collected. The magnitudes of the correlations between the physical functioning scale and the role functioning, pain, and fatigue scales were consistent with the values published in other studies across different geographical populations, further cross-validating the results from our study.

**Conclusion:**

The results from this study provide important first insights into using EORTC QLQ-C30 in the Kenyan population. We conclude that the questionnaire is an acceptable, reliable, and valid instrument for measuring the QOL in cancer patients in Kenya and recommend its use in clinical practice.

## Background

Cancer is the third leading cause of death in Africa, after infectious and cardiovascular diseases. The annual incidence of cancer in Kenya is about 28,000 new cases with an annual mortality of 22,000 cases, that is, 78.5% of the patients do not survive [21]. Of the cases reported, esophageal, cervical, breast, and prostate cancers were the leading causes of cancer mortality in the country [21].

Moi Teaching and Referral Hospital (MTRH) is the second-largest National Teaching and Referral Hospital (level 6 Public Hospital) in Kenya [10], treating cancer patients from the western half of the country as well as neighboring east African countries including Tanzania, Rwanda, Uganda, and South Sudan. Since opening in 2009, the Chandaria Cancer and Chronic Diseases Centre (CCCDC) clinic, located at MTRH, has seen over 13,000 cancer patients. Due to several challenges including lack of expertise at peripheral facilities, socio-economic limitations, as well as cultural beliefs and norms, many patients present with advanced disease, at which point, the impetus is on providing supportive and palliative care rather than treatment options.

Recognizing that cancer is a chronic condition, understanding the patient’s perception of their quality of life (QOL) has been shown to add value to the quality of care they receive by improving communication between health care professionals and patients [4, 18, 19], and ensuring continuity of care [6, 20] and appropriate symptom management. The importance of patient-reported outcomes in assessing their needs and outlook over the course of their cancer journey has been widely recognized in clinical practice. Further, the U.S. Food and Drug Administration (FDA) and European Medicines Agency (EMEA) now recognize the benefits of health-related QOL as a basis for approval of new anticancer drugs [5, 8], and many international research groups include QOL as a significant outcome measure in their clinical trials.

The EORTC-QLQ-C30 questionnaire was developed by the European Organization for Research and Treatment of Cancer (EORTC) over 25 years ago as an integrated measurement system for evaluating the quality of life patients participating in international clinical trials [1]. The questionnaire has been validated and is one of the most widely used cancer-specific instruments. Furthermore, the availability of an EORTC-translated version of the questionnaire in Kiswahili, one of the two official languages in Kenya, made the EORTC-QLQ-C30 an attractive tool to measure patient-reported outcomes in Kenyan cancer patients.

A comprehensive literature search revealed only a few instances of the use of QOL measures to evaluate cancer care in the sub-Saharan African region. Specifically, we did not encounter any published evidence of the use of QOL tools in the evaluation of cancer care in Kenya. Therefore, our primary objective was to evaluate the acceptability, reliability, and validity of the EORTC QLQ-C30 questionnaire in a cross-sectional sample of Kenyan cancer patients at the CCCDC clinic at MTRH.

## Methods

### Design and participants

This study was approved by the MTRH Institutional Research and Ethics Committee. Between July and August 2019, a heterogeneous sample of 100+ patients was recruited from the Oncology Clinic at CCCDC, associated with MTRH in Eldoret, Kenya. This represented 10% of the total number of patients seen per day at the clinic. The inclusion criteria for the subjects were a diagnosis of the most common types of cancer in Western Kenya (including but not limited to breast, cervical, prostate, lung, Kaposi sarcoma, esophageal, pancreatic, and colon) and age 18 years or older. Patients with hematological disorders were excluded from this initial cross-sectional study for logistical reasons. Clinicians were provided training on the purpose of the EORTC-QLQ-C30 questionnaire, its structure, and administration to patients. Participating subjects were selected using systematic sampling (every 7th patient) during scheduled consultation visits. After the clinical consultation, the patient was introduced to the questionnaire and its purpose was explained. Informed consent was obtained, following which, the patient was asked to complete the QLQ-C30 questionnaire. Clinicians were provided with official versions of QLQ-C30 both in English and Kiswahili (translated by the EORTC and obtained with permission), as both are official languages in Kenya. Patients were allowed to answer the questionnaire in their preferred language. For patients that were unable to read in either language, the questionnaire was administered by the clinician. In addition to the QLQ-C30, each questionnaire package included questions on demographics such as age and gender, and clinical variables such as performance status, type of cancer, disease stage, and type of treatment that were answered by the clinician. A post-administration clinician debrief was included to collect data on the time taken to complete the QLQ-C30 questionnaire and whether the questionnaire was self-administered or administered by the clinician. Additionally, data regarding whether patients found any questions confusing or difficult to answer were also collected in the clinician debrief. Clinicians were instructed to mark items that the patient asked for clarification on or indicated their inability to answer.

### Instrument

The EORTC QLQ-C30 (version 3) is a 30-item cancer-specific questionnaire for measuring the health-related quality of life (QOL) in cancer patients [1]. It includes five functioning scales (physical, PF; role, RF; cognitive, CF; emotional, EF; and social, SF), three symptom scales (fatigue, FA; pain, PA; and nausea and vomiting, NV), a global health status/QOL scale (GL), and six single items (dyspnea, appetite loss, sleep disturbance, constipation, diarrhea, and financial impact of the disease and treatment). All items employ a 4-point Likert scale, ranging from 1 (not at all) to 4 (very much), with the exception of two items in the GL scale, which use 7-point scales.

### Data analyses

Data analyses were performed using Stata (version 13) and Statistical Packages of Social Sciences (SPSS) software packages. Descriptive statistics were used to report demographic and clinical characteristics.

#### Acceptability

The acceptability of the EORTC-QLQ-C30 in our setting was assessed in terms of response rate, percentage of missing data, time needed to complete the questionnaire and items on the questionnaire that patients considered confusing or difficult to answer.

#### QOL assessment

The raw scores for the scales and single items were linearly transformed to values between 0 and 100 as described in the EORTC QLQ scoring manual [7]. The mean and standard deviation of each scale/single item were calculated. A higher score for a functioning scale represented a healthier level of functioning, a higher score for the global health status scale represented a higher QOL, and a higher score for a symptom scale/item represented a worse level of symptomatology.

#### Reliability

Reliability of a tool is primarily an indication of its ability to measure results in a consistent manner. The internal consistency of each scale of the questionnaire was assessed using Cronbach’s α-coefficient, where α-coefficient values ≥0.7 indicated adequate scale reliability of the tool [3, 15]. Since this study involved a one-time measurement only, other properties of reliability such as test-retest reliability and inter-rater reliability were not evaluated.

#### Validity

The validity of a measurement instrument is the degree to which the instrument measures the construct it purports to measure [14]. Construct validity was assessed by examining the correlations among subscales of EORTC QLQ-C 30 by Pearson’s correlation coefficient (r). Cross-cultural validity was evaluated by examining the number of missing records as well as the number and type of questions that participants found confusing or difficult to answer.

## Results

### Patient characteristics

Of the 110 filled questionnaires obtained from patients at the CCCDC clinic, 100 were considered evaluable for acceptability, reliability, and validity after verification of the questionnaires for completeness of essential data and accuracy. Patient demographics and clinical characteristics are detailed in Table 1. Of the 100 patients included in the study, 59 were female. The median age was 53.5 years (range 18–83). Breast cancer was the most prevalent type (40%), followed by prostate cancer (15%) and Kaposi sarcoma (10%). Although a majority (81%) of the patients were diagnosed with stage III/IV disease, 64% of the patients had a good performance status (ECOG-PS 0–1) [17]. Approximately 80% of the patients were on treatment (chemotherapy, radiation therapy, or surgery) at the time of data collection. Of the patients receiving chemotherapy, the majority had completed between 1 and 8 cycles of treatment, with a maximum of 14 cycles.

**Table 1:** Patient Demographics and Clinical Characteristics

	% (***N*** = 100)
**Age (years)**	
**Median**	53.5
**Range**	18–83
**Gender**	
**Female**	59
**Male**	41
**Primary cancer**	
**Breast**	40
**Prostate**	15
**Kaposi Sarcoma**	10
**Lung**	8
**Other**^a^	27
**ECOG-PS**	
**0–1**	64
**2**	7
**3–4**	4
**Unknown**	25
**Stage of disease**	
**I**	1
**II**	6
**III**	20
**IV**	61
**Unstaged**^b^	12
**Treatment**	
**Chemotherapy**	97
**Radiotherapy**	7
**Surgery**	1
**Patient status**	
**Existing**	79
**New**	21
**Number of chemotherapy cycles received (*N*** **= 73)**	
**Mean** ± **SD**	4.71 ± 2.88
**Range**	1–14

^a^Other cancers include colon, esophagus, pancreas, rectum

^b^ Includes Kaposi sarcoma patients, where there is no officially accepted system for staging (*n* = 10), and new patients that did not have their disease staged yet (*n* = 2)

### Acceptability

**Fig. 1:**
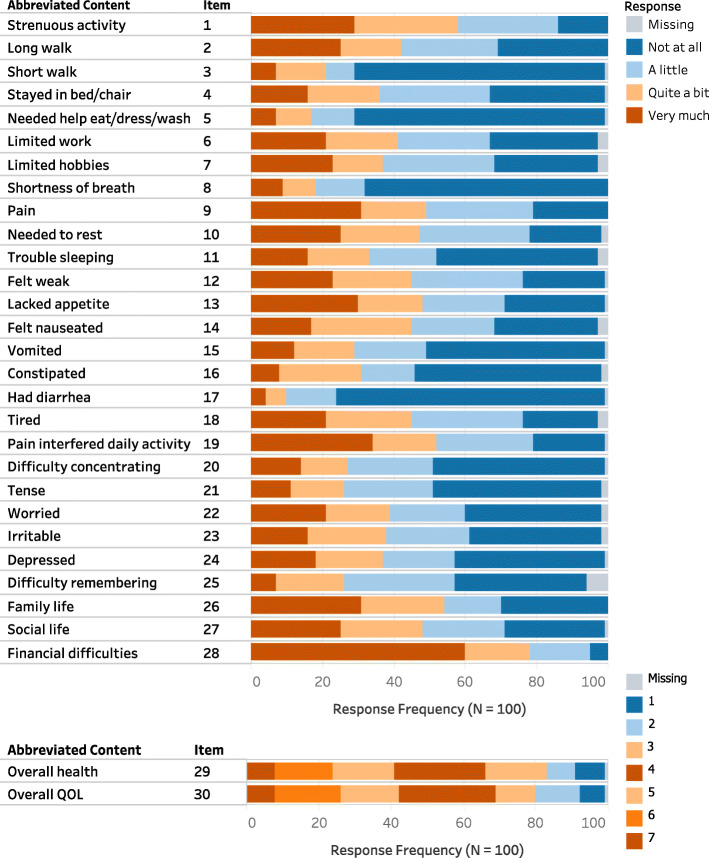
Percentage of missing values and frequency distribution of responses to the EORTC QLQ-C30. Items 1–28 have 4 response categories namely, “Not at all”, “A little”, “Quite a bit”, and “Very much”. Items 29–30, which are related to global health status and overall QOL, have response options between 1 and 7, where 1 is “Very poor” and 7 is “Excellent”

More than half the patients (*n* = 66) were able to self-administer the EORTC QLQ-C30 questionnaire, while the remaining 34 patients were administered the questionnaire interview-style by a clinician. The average time for completion of the questionnaire, whether self-administered, or interviewed by a clinician, was approximately 13 min (range 5–30 min), with interview-style questionnaires logically taking longer to complete than self-administered questionnaires. Item-level descriptive statistics are shown in Fig. 1. The rates of missing responses for each item were low (0–6%). The majority of the patients (71%) did not require any assistance with completing the questionnaire. Most patients also reported that the questions were clear and easy to understand while 5–9% of patients found at least one question confusing or difficult to answer (mainly items 21, 22, 24, and 30).

### QOL assessment

Mean scores for each scale of the QLQ-C30 are listed in Table 2. According to EORTC QLQ-C30, a high functional scale score represents a high/healthy level of functioning, while a high symptom scale score indicates a high level of symptomatology or problems [1]. The participants scored a global health status/QOL scale (GL) mean score of 53 (SD = 27). Functional scale scores ranged from 51 ± 36 for social functioning to 68 ± 28 for emotional functioning. Symptom scales ranged from 12 ± 24 for diarrhea to 79 ± 31 for financial difficulties.

**Table 2:** Scores and Cronbach’s Alpha-Coefficient Values for Each Scale/Item in the EORTC QLQ-C30

Scale/Items	Item No.^**a**^	Mean (SD)	Cronbach’s alpha
Global health status/QOL (GL)	29, 30	53 (27)	0.95
Functional Scales^b^			
**Physical (PF)**	1–5	63 (28)	0.86
**Role (RF)**	6, 7	55 (35)	0.87
**Emotional (EF)**	21–24	68 (28)	0.72
**Cognitive (CF)**	20, 25	63 (32)	0.37
**Social (SF)**	26, 27	51 (36)	0.75
Symptom Scales^c^			
**Fatigue (FA)**	10, 12, 18	49 (32)	0.85
**Nausea and vomiting (NV)**	14, 15	36 (34)	0.85
**Pain (PA)**	9, 19	54 (35)	0.83
**Dyspnoea (DY)**	8	19 (32)	–
**Sleep disorders (SL)**	11	35 (38)	–
**Appetite loss (AP)**	13	50 (39)	–
**Constipation (CO)**	16	30 (35)	–
**Diarrhea (DI)**	17	12 (24)	–
**Financial impact (FI)**	28	79 (31)	–

Cronbach’s alpha values > 0.70 indicate adequate scale reliability

*EORTC QLQ-C30* European Organization for Research and Treatment of Cancer QOL measure

^a^Item numbers on the QLQ-C30

^b^Scores range from 0 to 100, with a higher score representing a higher level of functioning

^c^Scores range from 0 to 100, with a higher score representing a higher level of symptoms

### Reliability

Cronbach’s α values are listed alongside mean scores for each scale of the QLQ-C30 in Table 2. All of the scales had a Cronbach α ≥ 0.70, with the exception of the cognitive function scale, CF (0.37).

### Validity

Table 3 shows correlations between subscales of EORTC QLQ-C30. The absolute magnitude of correlation coefficients ranged from 0.07 to 0.73 and most of the interscale correlations were significant at the 0.05 level. The general health/QOL subscale (GL) was correlated significantly with all other subscales; however, only role functioning, pain and fatigue were correlated at r ≥ 0.40. The highest positive and negative correlations were between the pain and fatigue (0.73) and role functioning and fatigue (− 0.69) subscales, respectively. In terms of convergent validity, all items showed a good correlation with their own scales (r ≥ 0.4) and were more highly correlated with their own scale than with the other scales.

**Table 3:** Interscale Correlations of the EORTC QLQ-C30

	PF	RF	CF	EF	SF	FA	NV	PA	DY	SL	AP	CO	DI	FI
**RF**	0.66													
**CF**	0.56	0.53												
**EF**	0.43	0.32	0.58											
**SF**	0.35	0.42	0.47	0.48										
**FA**	−0.63	**−0.69**	−0.66	−0.55	−0.40									
**NV**	− 0.23	− 0.40	− 0.38	− 0.39	− 0.28	0.52								
**PA**	−0.60	− 0.64	− 0.53	− 0.41	−0.51	**0.73**	0.41							
**DY**	−0.66	−0.48	−0.52	− 0.39	−0.36	0.51	0.32	0.41						
**SL**	−0.68	−0.57	−0.63	− 0.48	−0.46	0.68	0.41	0.67	0.66					
**AP**	−0.28	−0.46	−0.31	− 0.33	−0.14	0.61	0.65	0.44	0.24	0.39				
**CO**	−0.24	−0.25	−0.38	− 0.40	−0.35	0.46	0.43	0.45	0.27	0.35	0.42			
**DI**	−0.19	−0.21	−0.14	− 0.13	−0.11	0.25	0.32	0.17	0.24	0.15	0.19	0.26		
**FI**	−0.30	−0.38	−0.25	− 0.27	−0.46	0.39	0.24	0.29	0.15	0.25	0.20	0.07	0.09	
**GL**	0.35	0.42	0.20	0.20	0.35	−0.42	−0.29	−0.46	−0.22	− 0.36	−0.21	− 0.22	−0.22	− 0.34

Highest positive and negative correlations are in **bold**. Negative correlations are due to scoring procedures. *PF* Physical functioning, *RF* Role functioning, *CF* Cognitive functioning, *EF* Emotional functioning, *SF* Social functioning, *FA* Fatigue, *NV* Nausea and vomiting, *PA* Pain, *DY* Dyspnea, *SL* Sleep disorders, *AP* Appetite loss, *CO* Constipation, *DI* Diarrhea, *FI* Financial impact, *GL* Global quality of life, *EORTC QLQ-C30* European Organization for Research and Treatment of Cancer QOL measure

## Discussion

This cross-sectional study was designed to explore the acceptability, reliability and validity of using the EORTC QLQ-C30 questionnaire in Kenyan patients with heterogeneous types of cancer. Our results indicate that the instrument was relatively easy to use with high compliance and no major challenges. Patients were able to complete the questionnaire in a reasonable amount of time (average of 13 min), thus not adding a significant time burden to an already resource-limited setting. Patients were given the choice to complete the questionnaire in English or a validated translated version in Kiswahili - 64% of patients chose the English version while the remaining 36% completed the Kiswahili version. The overall low incidence of missing records indicates the cross-cultural validity of the translated version of EORTC QLQ-C30 in Kiswahili. Questions that were found confusing or difficult to answer (mainly items 21, 22, 24, and 30) may potentially be attributable to translation issues. For example, the phrases for “to feel tense” or “to be worried” may be used interchangeably in Kiswahili. Nevertheless, given the small number of patients (5–9%) that had difficulties in answering the questions, we do not recommend removal of any of the questions while using QLQ-C30 in our setting.

Preliminary QOL assessment from this cross-sectional study indicated an average QOL in Kenyan cancer patients (53 ± 27). Among the function scales, participants scored the lowest on the social function scale (51 ± 36) whereas among the symptom scales, participants scored the highest on the financial difficulties scale (79 ± 31). Neither of these results is surprising considering Kenya falls under the lower middle income category (data.worldbank.org) and is highly collectivistic, meaning its people are highly interdependent on their social and family circles. Nonetheless, the results provide supporting evidence for the notion that extending additional social support and financial aid may improve QOL outcomes in this patient population.

Reliability analysis, in terms of internal consistency, for multi-item subscales yielded similar results to previous studies in different populations [1, 2, 9, 11, 13]. These studies also noted a low Cronbach’s α-coefficient value for the cognitive functioning (CF) subscale, comparable to our findings (0.37). Although the CF subscale has poor psychometric properties, it is purported to include clinically relevant items [13] and therefore the construction of the scale and its inclusion in the tool is considered justifiable.

Validity testing of QOL measurements is becoming increasingly important, particularly since the FDA released its guidance on the use of patient-related outcome measures in medical product development to support labeling claims [8]. Pearson correlation analysis confirmed the satisfactory construct validity of the EORTC QLQ-C30 with most subscales being significantly correlated (*p* < 0.05). Similar to previous findings [2, 12], the general health/QOL scale (GL) displayed a modest correlation with the other subscales, with the exception of role functioning, pain, and fatigue, which displayed strong correlations. Furthermore, the magnitudes of the correlations between the PF scale and the RF, PA, and FA scales (all of which were strongly correlated) were consistent with the values from other studies [2, 9, 11, 13, 16, 22] across different geographical non-Western populations (Table 4), providing cross-validation for the results from this study.

**Table 4:** Comparisons of Correlation Coefficients Between the Physical Functioning scale and Each of the Role Functioning, Fatigue, and Pain scales Across Populations

	Kenyan (present study)	Korean [22]	Japanese [11]	Singaporean [13]	Turkish ([2, 9])
	**PF**	**PF**	**PF**	**PF**	**PF**
**RF**	0.66	0.68	0.83	0.44	0.63, 0.69
**FA**	−0.63	− 0.64	−0.61	− 0.61	−0.61, − 0.72
**PA**	− 0.60	−0.60	− 0.46	−0.60	− 0.46, − 0.50

*PF* Physical functioning, *RF* Role functioning, *FA* Fatigue, *PA* Pain

This study has certain limitations, for e.g., the sample size, while reasonable, may not be sufficient to make generalized statements regarding the robustness of the results and their applicability in patients with all types of cancer. In addition, the classification of the disease stage and performance status could not be obtained from all patients, therefore additional analyses evaluating potential correlations between these patient parameters and QOL scores could not be conducted. Nevertheless, the results from this study provide important first insights into using EORTC QLQ-C30 in the Kenyan population.

## Conclusion

The present study indicates that EORTC QLQ-C30 version 3.0 is a reliable and valid instrument and suitable for measuring the QOL in cancer patients in Kenya. As shown by this study, the majority (81%) of cancer patients referred to MTRH present with late-stage disease (stages III and IV), where they greatly benefit from palliative care, counselling, and end of life care. It is critically important to hear the patients’ voice throughout these processes to allow for improvement of the design and evaluation of interventions, and possibly enable patient-tailored intervention. Therefore, we recommend the integration of the EORTC-QLQ-C30 measure in the provision of palliative care to cancer patients in the Chandaria Center for Cancer and Chronic Diseases.

## Data Availability

The datasets used and/or analysed during the current study are available from the corresponding author on reasonable request.

## References

[1] Aaronson, N. K., Ahmedzai, S., Bergman, B., Bullinger, M., Cull, A., Duez, N. J., et al. (1993). The European Organization for Research and Treatment of Cancer QLQ-C30: A quality-of-life instrument for use in international clinical trials in oncology. *Journal of the National Cancer Institute*, *85*(5), 365–376. 10.1093/jnci/85.5.365. 10.1093/jnci/85.5.365 8433390

[2] Cankurtaran ES, Ozalp E, Soygur H, Ozer S, Akbiyik DI, Bottomley A (2008). Understanding the reliability and validity of the EORTC QLQ-C30 in Turkish cancer patients. European Journal Cancer Care (England).

[3] Cronbach LJ, Warrington WG (1951). Time-limit tests: Estimating their reliability and degree of speeding. Psychometrika.

[4] Detmar SB, Muller MJ, Schornagel JH, Wever LD, Aaronson NK (2002). Health-related quality-of-life assessments and patient-physician communication: A randomized controlled trial. JAMA.

[5] European Medicines Agency (2016) Appendix 2 to the guideline on the evaluation of anticancer medicinal products in man. https://www.ema.europa.eu/en/documents/other/appendix-2-guideline-evaluation-anticancer-medicinal-products-man_en.pdf.

[6] Fallowfield L (2002). Quality of life: A new perspective for cancer patients. Nature Reviews. Cancer.

[7] Fayers, P., Aaronson, N., & Bjordal, K. (1999). *EORTC QLQ-C30 scoring manual*, (2nd ed.) Brussels: EORTC Quality of Life Study Group.

[8] FDA Center for Drug Evaluation and Research (2009) Patient-reported outcome measures: Use in medical product development. https://www.fda.gov/media/77832/download.

[9] Guzelant A, Goksel T, Ozkok S, Tasbakan S, Aysan T, Bottomley A (2004). The European Organization for Research and Treatment of Cancer QLQ-C30: An examination into the cultural validity and reliability of the Turkish version of the EORTC QLQ-C30. European Journal Cancer Care (England).

[10] MTRH Hospital (2019). http://www.mtrh.go.ke/. Accessed 22 Aug 2020.

[11] Kobayashi, K., Takeda, F., Teramukai, S., Gotoh, I., Sakai, H., Yoneda, S., Noguchi, Y., Ogasawara, H., Yoshida, K. (1998). A cross-validation of the European Organization for Research and Treatment of Cancer QLQ-C30 (EORTC QLQ-C30) for Japanese with lung cancer. *European Journal of Cancer*, *34*(6), 810–815. 10.1016/s0959-8049(97)00395-x. 10.1016/s0959-8049(97)00395-x 9797690

[12] Kuenstner S, Langelotz C, Budach V, Possinger K, Krause B, Sezer O (2002). The comparability of quality of life scores. A multitrait multimethod analysis of the EORTC QLQ-C30, SF-36 and FLIC questionnaires. European Journal of Cancer.

[13] Luo N, Fones CS, Lim SE, Xie F, Thumboo J, Li SC (2005). The European Organization for Research and Treatment of Cancer quality of life questionnaire (EORTC QLQ-c30): Validation of English version in Singapore. Quality of Life Research.

[14] Mokkink, L. B., Terwee, C. B., Patrick, D. L., Alonso, J., Stratford, P. W., Knol, D. L., Bouter, L. M., de Vet, H. C. (2010). The COSMIN study reached international consensus on taxonomy, terminology, and definitions of measurement properties for health-related patient-reported outcomes. *Journal of Clinical Epidemiology*, *63*(7), 737–745. 10.1016/j.jclinepi.2010.02.006. 10.1016/j.jclinepi.2010.02.006 20494804

[15] Nunnally, J. C., & Bernstein, I. H. (1994). *Psychometric theory. McGraw-hill series in psychology*, (3rd ed.) New York: McGraw-Hill.

[16] Silpakit C, Sirilerttrakul S, Jirajarus M, Sirisinha T, Sirachainan E, Ratanatharathorn V (2006). The European Organization for Research and Treatment of Cancer quality of life questionnaire (EORTC QLQ-C30): Validation study of the Thai version. Quality of Life Research.

[17] Sørensen JB, Klee M, Palshof T, Hansen HH (1993). Performance status assessment in cancer patients. An inter-observer variability study. British Journal of Cancer.

[18] Takeuchi, E. E., Keding, A., Awad, N., Hofmann, U., Campbell, L. J., Selby, P. J., Brown, J. M., Velikova, G. (2011). Impact of patient-reported outcomes in oncology: A longitudinal analysis of patient-physician communication. *Journal of Clinical Oncology*, *29*(21), 2910–2917. 10.1200/JCO.2010.32.2453. 10.1200/JCO.2010.32.2453 21690465

[19] Velikova G, Booth L, Smith AB, Brown PM, Lynch P, Brown JM, Selby PJ (2004). Measuring quality of life in routine oncology practice improves communication and patient well-being: A randomized controlled trial. Journal of Clinical Oncology.

[20] Velikova, G., Keding, A., Harley, C., Cocks, K., Booth, L., Smith, A. B., Wright, P., Brown, J. M. (2010). Patients report improvements in continuity of care when quality of life assessments are used routinely in oncology practice: Secondary outcomes of a randomised controlled trial. *European Journal of Cancer*, *46*(13), 2381–2388. 10.1016/j.ejca.2010.04.030. 10.1016/j.ejca.2010.04.030 20570138

[21] Wambalaba, F. W., Son, B., Wambalaba, A. E., Nyong'o, D., & Nyong’o, A. (2019). Prevalence and capacity of cancer diagnostics and treatment: a demand and supply survey of health-care facilities in Kenya. *Cancer Control*, *26*(1), 1073274819886930. 10.1177/1073274819886930. 10.1177/1073274819886930 PMC6893940 31795739

[22] Yun, Y. H., Park, Y. S., Lee, E. S., Bang, S. M., Heo, D. S., Park, S. Y., You, C. H., West, K. (2004). Validation of the Korean version of the EORTC QLQ-C30. *Quality of Life Research*, *13*(4), 863–868. 10.1023/B:QURE.0000021692.81214.70. 15129896

